# Temporal Modulation of Drug Desensitization Procedures

**DOI:** 10.3390/cimb44020057

**Published:** 2022-02-08

**Authors:** Razvan Costin Stan

**Affiliations:** Department of Biomedical Sciences, Chonnam National University, Gwangju 61469, Korea; strazvan@jnu.ac.kr; Tel.: +82-062-220-6744

**Keywords:** drug desensitization, circadian clock, molecular memory, IgE, allergen

## Abstract

Drug hypersensitivity reactions are an unavoidable clinical consequence of the presence of new therapeutic agents. These adverse reactions concern patients afflicted with infectious diseases (e.g., hypersensitivity to antibiotics), and with non-infectious chronic diseases, such as in cancers, diabetes or cystic fibrosis treatments, and may occur at the first drug administration or after repeated exposures. Here we revise recent key studies on the mechanisms underlying the desensitization protocols, and propose an additional temporal regulation layer that is based on the circadian control of the signaling pathway involved and on the modulation of the memory effects established by the desensitization procedures.

## 1. Introduction

Drug desensitization procedures (DS) induce a refractive, hypo-sensitive but temporary response state to an offending drug. For some patients, the allergenic drug may be essential to therapy and avoidance will lead to impaired drug management and reduced life expectancy [1]. Despite the medical implications, only empirical protocols are available to reduce the effect of full dose administration of the offending drug, by using single or multiple sub-optimal doses, with the aim to diminish its allergenic potential. DS relies on the establishment of short-term memory only to particular rates and dose/concentrations of the drug administration protocol [2]. Both IgE and non-IgE mediated hypersensitivity reactions (HSR) have been described for a wide range of antigens, with the former being more prevalent and more widely studied. DS is performed in patients with IgE-mediated reactions; protocols are also available in reactions where drug-specific IgE have not been demonstrated [3]. While the clinical emphasis has been mostly focused on dosage, temporal aspects involved in the desensitization protocols in IgE-mediated hypersensitivity reactions have not been addressed in detail. Therefore, it is timely to address the relevance of circadian clocks to the clinical use of DS protocols, in order to optimize the duration of clinical effects and facilitate therapy. This review will present the main events involved in the regulation of mast cells, followed by an overview of the drug desensitization procedures. The temporal regulation of these procedures will be emphasized with regards to (1) their algorithmic nature and (2) the influence of the circadian clocks in mast cells and basophils on successful desensitization outcomes.

## 2. Drug Desensitization: Positive and Negative Role of Mast Cells

Mast cells (MCs) are essential effectors in IgE (immunoglobulin E)- and non-IgE-mediated HSR and constitute the primary cellular target for desensitization (DS) procedures. Tissue MCs become nonreactive to the drug of interest, after desensitization has been successfully achieved [4]. While the subsequent administration of suboptimal doses of antigen prior to the optimal dose renders these cells unresponsive to the drug of interest, DS is not achieved for other stimuli [5]. The IgE-mediated pathway is better understood in these cells, and encompasses an initial class switch in allergen specific antibodies from IgM to IgE, after exposures have occurred [6]. The allergen-specific IgE binds to its Fc receptors (FcεRI) with very high affinity (K_D_ of 10^−10^ M as measured with surface plasmon resonance to a higher limit K_D_ of 10^−12^ M when assessed with differential scanning calorimetry) [7]. The FcεRI are heterotetramer receptors composed of α, β, and two γ chains that are constitutively expressed on basophils and in human MC. To recognize the allergen in complex with the IgE, the α chain uses two extracellular Ig-like domains; β and γ chains are responsible for intracellular signal transduction via a single immune-receptor tyrosine-based activation motif (ITAM). Phosphorylation of the ITAM γ-unit is responsible for initiating the signal, and phosphorylation of the β-unit may have a suppressor function, the latter effect mediated by a Lyn tyrosine kinase that is constitutively associated with it [8]. Upon allergen ligation to the IgE-bound FcεRI, the formation of FcεRI cross-links occurs, a key event that will trigger the downstream activation of Lyn and phosphorylation of FcεRI. After phosphorylation, Syk tyrosine kinase is recruited to phosphorylated γ chain from ITAM and phosphorylates other signaling proteins from the signaling cascade, including LAT1 (linker for activation of T cell). LAT1 will further activate PLCγ-2 (phospholipase C Gamma) that helps hydrolyze cell membrane PIP2 (phosphatidylinositol 4,5-bisphosphate) into IP3 (inositol 1,4,5-trisphosphate) and DAG (diacylglycerol). The resulting IP3 binds to IP3 receptors residing on the endoplasmic reticulum (ER) that will mediate Ca^2+^ release from this organelle, an important trigger for the exocytotic release of inflammatory mediators. In turn, DAG interacts with DAG-dependent isoforms of cytosolic PKC (protein kinase C) that will regulate the release of mediator-containing granules. This event may occur due to the phosphorylation of the myosin light chain and the control of actin polymerization, a necessary step for degranulation (release of granules) [8]. Calcium depletion from the ER sites is detected by ER calcium-sensor STIM-1 (stromal interaction molecule), that will diffuse to the plasma membrane and form contact points with calcium channel proteins Orai1 and Transient receptor potential canonical (TRPC). These structures will mediate extracellular Ca^2+^ influx so as to replenish the ER Ca^2+^ pool and thus further sustain calcium-dependent intracellular signaling, as long as IP3 is produced [8]. Degranulation involves signal coordination between associated cytoskeletal reorganization and the membrane fusion machinery that enables lipid bilayer mixing and the subsequent exocytosis, with actin reorganization directly dependent on calcium influx [5]. Elevated signals of intracellular calcium are thus essential for MC activation and for the release of granules containing a host of preformed inflammatory molecules, including histamine, cytokines, leukotrienes and heparin.

**Figure 1:**
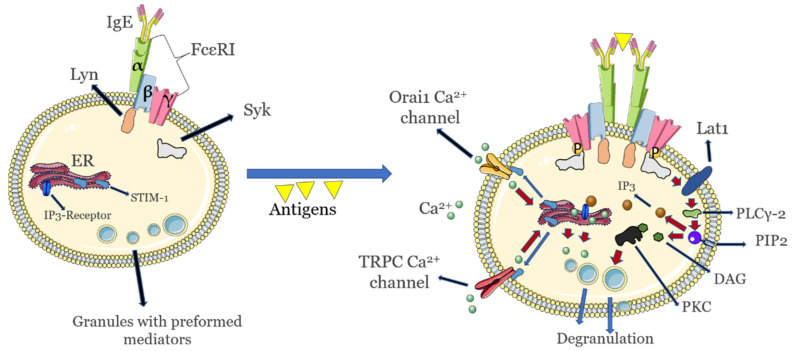
Key initial activation and signaling events in allergic (IgE)-mediated sensitivity and hypersensitivity to allergens in mast cells. Endoplasmic reticulum release of calcium that is important for degranulation is highlighted. Calcium release from the Golgi apparatus that also takes part in mast cell degranulation is not shown. Images not to scale.

An overview of these events is shown in Figure 1 below where only the initial sensitization and degranulation events are presented.

The net result of these signaling events is diachronically different: an immediate release (within minutes) of an array of inflammatory mediators, including amines (e.g., histamine), cytokines, β-hexosaminidases and growth factors from cytoplasmic granules is followed by a late release of inflammatory cytokines, around 4–6 h after the initial FcεRI cross-linking [9].

In order to dampen the MC signaling and induce DS, negative regulation occurs at multiple levels. One such mechanism relies on the co-aggregation of FcεRI with the low-affinity IgG receptor FcγRIIB. This process requires IgG containing immune complexes that can cross-link FcγRIIB to IgE-loaded FcεRI or FcεRI-IgE-Ag complexes in stimulated mast cells [10]. Importantly, FcγRIIB also binds to different IgG isotypes that are responsible for decreasing the symptoms of IgE-based allergies, and are necessary to prevent and sustain immunological tolerance [10]. As a consequence of FcγRIIB-IgG activity, impaired antigen/IgE/FcεRI complex internalization may subsequently be in place [11]. Challenging with the same antigen at a later time without eliciting an immune response is a hallmark of DS, however different antigens, including those that induce calcium fluxes needed for signaling will still elicit an allergenic response [12]. Such a feature implies that DS is antigen-specific and FcεRI-IgE complexes are still available for binding other antigens (epitope spreading) and for subsequent cross-linking [13]. The MC refractory state to allergens may be due to the internalization of FcεRI-IgE complexes through progressive cross-linking that corresponds to increasing antigen concentrations [14], which could potentially limit the pool of allergen sensors. However, such a mechanism is not entirely accurate, as other studies indicate that functional FcεRI-IgE complexes may still remain on MC surfaces during DS and may still relay allergen signals intracellularly [15]. DS is not maintained because excess soluble antigen is present, as washed and re-challenged cells remain desensitized if the procedure is performed within 1.5 to 5 days [16,17]. The size of the antigen-IgE-FcεRI clusters may change as additional antigens are added, with corresponding changes in mobility on the cell surface, which may influence subsequent internalization and mediator release [18]. These events must be exquisitely regulated, as studies of human basophils show that as few as 50 of such stable aggregates can induce the release of mediators [19]. Such a low number is also encountered in the formation of immunological synapses, where only 10 to 100 cognate peptide–MHC complexes, from a total pool of 10,000–100,000 MHC molecules expressed on an antigen presenting cell, have to be recognized by T-cell receptors to generate an immune response, due to these receptors’ ability to be re-activated multiple times [20]. It is unclear whether the density of these antigen-IgE-FcεRI clusters bears any linear relation to the use of sub-therapeutic dose or doses employed during DS, or whether non-linear synergetic effects can be obtained by the cross-linking events. However, once DS has been established, disaggregating the cross-links between the antigen-IgE-FcεRI complexes has no effect on recovery from DS, as recently shown [21]. It has been proposed that the DS hallmark of increasing the sub-therapeutic doses provides sufficient amounts of antigens to bind to IgE, but not to cross-link them [22]. Alternatively, low doses of antigen may rearrange the cell membrane in antigen-sensitized MCs, preventing the internalization of the antigen/IgE/FcεRI complex and thus protecting against anaphylaxis [23]. At the same time, IgE binding to FcεRI leads to upregulation of FcεRI expression in MCs that will traffic to the cell membrane in vivo [24], in a process that does not depend on new protein synthesis [25]. Furthermore, in human basophils, removal of IgE from IgE-FcεRI complexes results in an accelerated loss only in the unoccupied FcεRI, with no effect on occupied receptors that are involved in DS [26]. Differences between the FcεRI expression in different cell lines are not related to FcεRIH gene copy numbers, or to differences in their steady state mRNA levels [27], hinting at regulation occurring strictly at the level of the IgE-FcεRI complex lifetime. Mast cells’ FcεRI not occupied by IgE has a half-life of around 24 h in vitro, while FcεRI bound to IgE appears to be permanently expressed [28]. Moreover, incubation of MCs with IgE appears to extend cell survival by modulating apoptosis [29], notwithstanding the fact that the IgE concentrations required for this effect are orders of magnitude higher than those necessary for allergen desensitization [30].

Downstream of these events, in vitro desensitization of human MCs decreases the expression levels of signal transducing molecules, such as Syk and Lyn [31]. Further, the levels of inflammatory mediators in desensitized MCs were shown to be intact following administration of non-desensitizing antigens [32]. It is possible that small amounts of degranulation may occur in some patients during DS and that low levels of histamine released in vivo would be rapidly metabolized, perhaps minimizing the clinical response, while in in vitro studies, the released β-hexosaminidase will accumulate [33]. Recent studies have also involved aberrant remodeling of actin during DS that negatively regulates calcium mobilization and prevents mediator release [34], and when receptors were desensitized through repeated stimulation with increasing doses of antigen, dynamic reorganization of the actin cytoskeleton is inhibited [35]. However, contradictory results were obtained during DS on basophils, where actin polymerization was shown to not play a role [36].

An involvement of the progressive internalization of G-protein coupled receptors (GPCR) upon binding to increasing amounts of released inflammatory modulators such as histamines, has been proposed as a model for the extended memory to DS doses [37]. Nonetheless, MC stimulation with low amounts of leukotrienes causes hyper-responsiveness to leukotriene stimulation due to receptor trafficking to the MC surface, while use of high leukotriene concentrations (by a factor of 1000) will lead to their receptor being internalized and subsequent MC hypo-responsiveness [38]. It is possible that in the former case, DS need not implicate receptor endocytosis but may rely on establishing a refractory state to binding at the level of individual GPCRs still localized on cell membranes [39]. On the other hand, in the latter case, trapping the ligand with the receptor by co-internalization into endosomes may be a means to preserve the molecular memory of the interactions and thus continue signaling [40].

It is evident that a single unifying mechanism behind DS has not yet been established, and that DS is regulated at different levels occurring on multiple time scales. The only constant feature of all DS procedures is the administration of an initial suboptimal dose, which can be either orders of magnitude lower than the final therapeutic dose, or in between this range. Following this key event, the allergen detection system is temporarily unable to properly gauge the increasing concentrations of the offending drug or allergens. This phenomenon may be analogous to aliasing, the erroneous detection of high frequency signals as low frequency signals, an inevitable feature in signal transfer processes.

We hypothesize that the persistence of the molecular memory underlying DS is a temporal feature of the *entire* MC signaling cascade that relies on all the signaling proteins involved relaxing back to partner-competent conformations. This hierarchy of timescales may be superimposed by the presence of a circadian clock, as discussed in the next section. As noted with the β-arrestins temporarily maintaining their active conformation after having dissociated from the GPCR [40] or with single enzymes [41], these timescales range from minutes to hours, and have been recently implicated in an experimental work of IgE-receptor stimulation [34]. In particular, this hypothesis would predict that the IgE-FcεRI complex could retain allergen specific conformations after allergen dissociation and processing. Indeed, instances have been documented where allergens are not needed to induce an allergic response, with so-called “cytokinergic” IgE being solely responsible for the activation of MC in the absence of allergen [30]. Absence of any allergenic activity was present in the MC purified IgE aggregates, as well as in the adventitious formation of IgE aggregates from monomeric IgE ex vivo that had not been stimulated [42,43,44]. Due to 1:1 stoichiometry between the IgE and FcεRI, antigen cross-linking will bring together and stabilize multiple FcεRI, a mechanism that is known to memorize signaling-competent conformations [41].

## 3. Temporal Aspects of DS Procedures

Hypersensitivity reactions (HSR) can occur with most drugs, may affect any organ, and vary greatly in severity from mild discomfort to life threatening anaphylaxis. In cases where the drug administration cannot be discontinued, drug DS must be performed by administering increasing doses of the medication until the final therapeutic dose is reached. The cumulative dose then becomes protective against anaphylaxis and it is memorized for a variable duration, until sensitization reoccurs. It is important to note in this context that patients who undergo successful rapid drug desensitization may still experience HSR even days later after concluding the treatment, to the same dose of the allergen that had just been tolerated, highlighting the temporary nature of DS as opposed to true clinical tolerance [45]. Importantly, low amounts of histamine were measured in desensitized MCs after as few as 24 h following DS [46,47]. At the same time, sensitization can also be immediate, if a dose higher than the desensitized dose is introduced at any time. It is important to note that if no antigen exposure occurs, an allergic reaction may be present to the same dose that had been previously tolerated [48]. Higher doses and/or sub-optimal durations of drug administration during DS are key risk factors for HSR.

### 3.1. Circadian Clock Modulation of IgE Reactions

**Figure 2:**
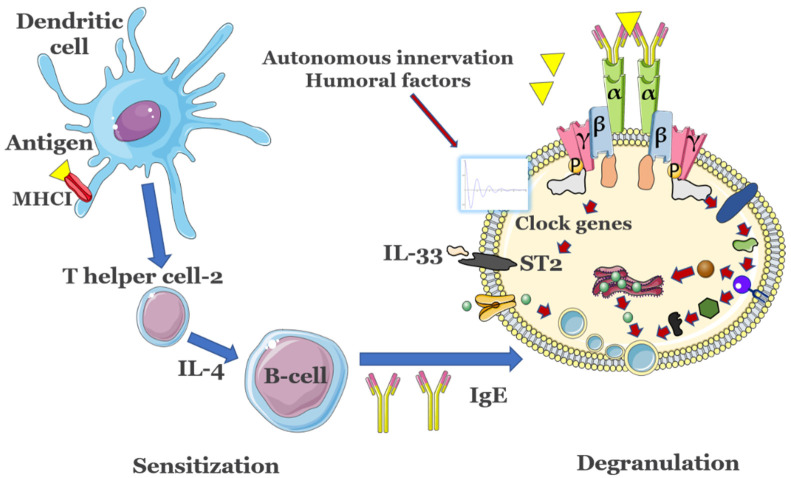
Key immune response events associated with the presence of allergens. Antigens are presented by major histocompatibility complex (MHC) class I molecules residing on membranes of dendritic cells (DCs) and, among other effects such as proliferation of antigen-specific cytotoxic CD4+ T helper cells, also lead to the production of innate cytokines. The latter modulate the changes in phenotype of DCs, promote development of T helper cells 2 (Th2) and will activate MCs and basophils. B cells promote IgE release after stimulation from TH2. In MCs, clock genes are modulated by a series of Zeitgebers to control the expression of suppressor of tumorigenicity 2 (ST2) or of FcεRIβ, modulating the allergic response. Importantly, activity of T cells and B cells is also controlled by their own internal circadian clocks. The detailed activation of mast cells is presented in Figure 1. Figure not to scale.

The circadian nature of IgE-mediated allergic diseases has been documented in, e.g., allergic rhinitis and asthma, where inflammatory activity becomes more pronounced in the early morning and at midnight [46]. The sputum from asthma patients revealed higher lymphocyte, neutrophil, and eosinophil counts in the early morning [49], compared to other times of the day, accompanied by increased serum pro-inflammatory IL-5 concentrations early in the morning [50]. Free IgE is constantly present in serum in considerable amounts even in the absence of antigen, with a half-life of 2 days in humans that does not appear to be controlled by a circadian clock [51]. In patients with allergies or atopic diseases, nonetheless, serum IgE levels are markedly increased [52]. In order to keep the number of unoccupied IgE receptor sites at a setpoint, MC may regulate FcεRI expression, possibly in response to the levels of circulating IgE. However, other components of the signaling machinery are expressed or activated in a circadian manner and may influence the allergenic response, as presented below. Circadian variations in, e.g., histamine release or in pro-inflammatory cytokine expression such as IL-6/IL-13 occur in MCs following IgE-mediated activation [53]. At the same time, IgE-mediated FcεRI up-regulation has been shown to augment the ability of MCs to release pro-inflammatory cytokines such as IL-4 [54]. IL-4 in turn enhances the expression of PER2 by a factor of 3–4 at the acrophase of the MC circadian rhythms [55]. IL-33 can activate both MCs and basophils by means of the receptor ST2. CLOCK, another important clock gene, can gate the MC and basophil response to IL-33 by regulating the rhythmic changes in ST expression [52]. CLOCK also promotes expression of FcεRIβ, an amplifier of FcεRI expression and its downstream signaling [53]. Circadian regulation of MC degranulation can also be modulated by the rhythmic expression of FcεRI [56]. As mentioned, free FcεRI has a half-life of around 24 h in vitro, while FcεRI bound to IgE appears to be permanently expressed [28]. FcεRI signaling is reduced when the core clock protein PER2 is highly expressed in MCs, likely by inhibition of CLOCK/BMA1 activity that directly affects expression of the beta subunit of FcεRI [54]. It is also conceivable that some HSR may occur when the MC circadian clock is blunted and administration of doses occurs at the acrophase of PER2 expression. In support of this hypothesis, wild-type bone marrow-derived mast cells overexpressing PER2 exhibited diminished cell-surface FcεRI expression and IgE-mediated intracellular Ca^2+^ mobilization, and reduced degranulation when compared with control cells [54]. Daily variations in the FcεRI levels might modulate the formation and duration of the FcεRI-IgE-antigen complexes, and thus the efficacy of subsequent DS dosing. Indeed, gating of circadian rhythms in MCs appears to be strongly influenced by PER2 levels that control the rhythmic secretion of corticosterone, that can further downregulate FcεRI expression in MC and suppress subsequent IgE-mediated reactions both in vivo and in vitro [57]. An overview of these key relations between the circadian clock and allergic reactions is shown in Figure 2.

Importantly, other clock genes are also activated in MCs and basophils, such as Bma1, Per1, Rev-erbα or Dbp, which respond to glucocorticoids or cortisone and control the release of leukotrienes or reset the circadian clock [53]. Pharmacological use of glucocorticoid receptor agonists, coupled with judicious administration times in an asthma model, were shown to minimize phase shifting in the expression of key clock genes, and thus of their clock-dependent proteins [58], providing an avenue to couple DS to circadian control of MC activity. It is important to stress that administration in vivo of such clock modifiers still necessitates a minimum of 2 h for the PER2 upregulation to be observed [59]. Pharmacological resetting of the circadian clock in mast cells and basophils to suppress IgE-mediated allergic reactions has also been demonstrated for compounds such as aminophylline or PF670462 in both animal models and patients [54]. Further downstream in the signaling cascade, the release of both pre-stored histamine and de novo synthesis of leukotrienes was observed to be modulated by a circadian clock [60].

### 3.2. Time Interval Control in DS Steps

An essential DS feature is that the allergen must be present during a critical period of time for desensitization to take place. Similar to pharmacological receptors where partial agonists cause less desensitization [61], different antigens might activate the IgE-FcεRI complexes with different efficacies. As such, the rapid administration of suboptimal doses must span between minutes to hours [62], although the risk of introducing a lethal medication remains high [4,63]. Although the empirical DS protocols vary widely in concentrations and spacing in time of doses, some key features can be outlined.

#### 3.2.1. DS Is Directly Dependent on the Interval between Doses

Evidence indicates that very small differences (i.e., minutes) between doses can have large effects on the activation of basophils and MCs, and on subsequent DS procedures. In some extreme cases in vitro, even 2 min were enough to observe noticeable changes in DS efficacy [64]. Conversely, the very last step of the DS during rapid desensitizations can be accelerated such that patients spend less time in intensive care or outpatient units [65].

#### 3.2.2. Modulation of the Intervals between Doses

Increasing the time interval between doses improves DS efficacy [66]. In cases where rapid desensitization is warranted, this time management may be more difficult to implement, although for many DS procedures, a minimum of 4 h has been empirically recommended. A superior limit for treatment is also warranted, as most basophils have a half-life of 12 h [67], and some extended protocols that breach this limit may have a HSR of 25% [68].

#### 3.2.3. Unique Dose vs. Sequential Desensitization

Suboptimal DS Protocol has been consistently shown to be not as effective as the gradual additions of the drug of interest [69].

#### 3.2.4. Temporal Modulation of DS Procedures May Be Just as Important as the Amount of the Administered Drug Ex Vivo

In some studies, increasing the concentration between doses by 5 or 10 folds did not alter the DS efficiency, whereas doubling intervals between DS steps (10 to 20 min) rather than halving them, reduced basophil activation by ~30% [70].

#### 3.2.5. Increasing the Dose Is Not as Effective as Increasing the Number and Duration of the Intervals

In instances where allergen concentrations were varied from 2 to 10 folds, the DS outcome is not affected [71]. In particular, such a property of the DS procedures implies that some patients could tolerate an accelerated dosing regimen, including in regimens administered according to a logarithmic algorithm [72]. Once a rapid DS protocol has been established, a second cycle of dosing may provide a much lower breakthrough-reaction prevalence than the first administration course [73]. For DS procedures against monoclonal antibodies, the most commonly used protocol has 12 steps but is associated with an increased risk of HSR during the very last dose administration; addition of a 13th step in the protocol seems safe and more effective [74]. A summary of the key dosage features is presented in Table 1 below.

It is important to note that mutations or changes in the activity of the clock genes may also underlie the unsuccessful DS procedures in some patients. For instance, mutations in the clock genes may affect histamine transport and aberrant non-circadian plasma histamine levels [75], as opposed to peak levels in both healthy and asthmatic patients in the early morning [76]. Furthermore, the expression level and distribution patterns of clock gene products may be asymmetrically present even between symmetric cavities such as within nasal mucosa, or between healthy controls and allergic rhinitis patients [77]. Downregulated circadian clock genes have been associated with increased risk of asthma compared to levels in healthy individuals, and may serve as a diagnostic tool [78].

It is not explicitly mentioned in the clinical literature whether the DS protocols are designed so that the circadian aspects of the MC and basophils are accounted for. Within a clinical setting, control of the circadian clock can be achieved via modulation of zeitgebers (light or feeding). Whenever possible, drug administration at optimal time-of-day of the offending drug might ensure a more uniform response to desensitization procedures [79]. The amplitude of changes in the clock genes was shown to depend on the allergen administration times in allergic rodents, resulting in modulation of the immune responses, such as the inhibition of TH2 cell activity and alleviation of allergic reactions [80].

Furthermore, it is conceivable that, when subsequent DS cycles are performed on patients who have completed the full course, maintaining the same administration times, whenever possible, may also be a means to alleviate some of the HSR observed.

## 4. Conclusions and Outlook

Despite numerous empirical attempts, no formal DS algorithm exists so far for all classes of allergens used in clinical practice. Even within the same class, great variation can be found for different patient groups [81], and a successful DS session can still be unsuccessful or even potentially lethal, when tried subsequently [74]. Diurnal symptoms are modulated by the circadian clock observed in patients with allergic diseases such as asthma, allergic rhinitis, and chronic urticaria [82]. The importance of chronobiology is being increasingly recognized in protective or palliative care. For example, time of day administration of vaccines with a clear skewing towards early mornings has now emerged as an important parameter to better elicit an immune response against pathogens such as the influenza [83] or SARS-CoV-2 [84]. On the other hand, daily plasma fluctuations in the concentrations of the allergen or therapeutic agents (i.e., monoclonal antibodies) were also measured, with higher concentrations in the evenings compared to mornings, a fact that may influence the DS procedures [85].

A hypothetical optimized administration of a DS regimen, based on the data outlined in this work, for instance using monoclonal antibodies against various cancers, would rely on a (1) daily administration in the evenings, with (2) higher intervals between doses (e.g., 20 min) for a (3) maximum duration of 4 h, and with the last dose significantly larger than the preceding dose (4). As outlined above, timely use (i.e., hours before allergen administration) of glucocorticoid receptor agonists to reset expression of key clock genes such as PER2, and thus to modulate MC activity, may provide an additional layer of control to prevent HSR.

Experimental desensitization protocols that account for circadian patterns of expression and activity of the IgE-mediated signaling pathway components should be explored as an additional layer of HSR control. Furthermore, measurements of the half-lives of the main effectors of this pathway must be obtained in order to delineate and perhaps modulate the duration of allergen desensitization procedures.

## Figures and Tables

**Table 1:** Common dosage/interval features in DS procedures.

Interval between Doses	DS Efficacy
1–5 min	low [65]
10–15 min	high [66,68]
20 min	highest [70]
accelerated last step	high [74]
Number of doses	
single (suboptimal)	low [69]
multiple	high [69]
Duration of 1 DS cycle	
minutes (min. 30 min)	low [62]
hours (max. 4 h)	high [68]
Dosage	
geometric progression	high [70]
logarithmic progression (accelerated regimen)	high [72]
geometric progression, last dose much higher (accelerated regimen)	high [73]
